# Impaired Modulation of Corticospinal Excitability in Drug-Free Patients With Major Depressive Disorder: A Theta-Burst Stimulation Study

**DOI:** 10.3389/fnhum.2019.00072

**Published:** 2019-02-26

**Authors:** Philippe Vignaud, Caroline Damasceno, Emmanuel Poulet, Jérôme Brunelin

**Affiliations:** ^1^INSERM U1028, CNRS UMR5292, PSYR2 Team, Lyon Neuroscience Research Center, Université Claude Bernard Lyon 1, Lyon, France; ^2^Centre Hospitalier Le Vinatier, Bron, France; ^3^Department of Psychiatric Emergency, Edouard Herriot Hospital, Lyon, France

**Keywords:** major depressive disorder, transcranial magnetic stimulation, theta-burst stimulation, cortical excitability, neural plasticity

## Abstract

Impaired neural plasticity may be an important mechanism in the pathophysiology of major depressive disorder (MDD). Coupled with electromyography (EMG), repetitive transcranial magnetic stimulation (rTMS) is a useful tool to evaluate corticospinal excitability and cortical neuroplasticity in living humans. The goal of this study was to compare rTMS-induced cortical plasticity changes in patients with MDD and in healthy volunteers. In this single-blind controlled study, 11 drug-free patients with MDD and 11 matched healthy controls were analyzed. Cortical excitability, measured by the amplitude of motor evoked potentials (MEPs) evoked by single-pulse TMS, was assessed before and repeatedly after (for 30 min) participants received a single session of intermittent theta-burst stimulation (iTBS) and continuous TBS (cTBS). rTMS was applied over the left motor cortex using a neuronavigation system. Intensity was set at 80% of the active motor threshold (AMT). A large interindividual variability was observed after both iTBS and cTBS in the two groups. At the group level, we observed impaired iTBS-induced neuroplasticity in patients with MDD compared to that in controls. No differences were observed between the groups regarding cTBS-induced neuroplasticity. Our results suggest impaired long-term potentiation (LTP)-like mechanisms in MDD.

**Clinical Trial Registration**: www.Clinicaltrials.gov, identifier #NCT02438163.

## Introduction

Unipolar major depressive disorder (MDD) is a very frequently occurring disorder associated with high impairment of global functioning and significant societal economic burden (Kessler et al., 2003; Whiteford et al., 2013). Despite current efforts, the pathophysiology of MDD is not completely elucidated. Among the several theories that have been proposed, the “neural plasticity abnormalities” theory in particular, which may bridge the prevailing theories, has gained attention (Wainwright and Galea, 2013). Neural plasticity encompasses an array of key brain mechanisms (birth, survival, migration, and integration of neurons, synaptogenesis and apoptosis). Several studies have reported impaired neural plasticity at different levels in patients with MDD. For instance, postmortem studies have revealed a reduction in the number of synapses and a decreased expression of synaptic function-related genes in the prefrontal cortex (PFC) of patients with MDD (Kang et al., 2012). Patients with MDD also display a reduction in brain volume compared with healthy volunteers, especially in the hippocampus (Campbell et al., 2004) and in the PFC (Drevets, 2000). Taken together, these studies suggest a close relationship between abnormal neural plasticity and MDD, but more studies are needed to establish the key role of these mechanisms in MDD pathophysiology (for a review see Cantone et al., 2017).

Transcranial magnetic stimulation (TMS) is a noninvasive brain stimulation method that can be used to evaluate some indexes of neural plasticity in living humans. Some authors have suggested that the modulation of motor corticospinal excitability measured by single pulse TMS following a repetitive TMS (rTMS) session may reflect neural plasticity (Cantone et al., 2017). Among all the currently available rTMS protocols, the theta burst stimulation (TBS) is a brief rTMS protocol enabling an assessment of cerebral plasticity, especially at a synaptic level (Huang et al., 2005). Depending on the stimulation parameters, TBS can induce either an inhibition of corticospinal excitability (following continuous TBS, cTBS), or an enhancement (following intermittent TBS, iTBS). In these studies, the induced modulation of corticospinal excitability is assessed by the size of motor evoked potentials (MEPs) measured before and after the rTMS session. For instance, by measuring the modulatory effects of a single TBS session in individuals with Asperger’s syndrome, Oberman et al. (2012) observed a significant alteration in the modulation of corticospinal excitability in patients compared to that in healthy volunteers in response to both iTBS and cTBS suggesting aberrant mechanisms of plasticity in patients. These results suggest that TBS may reveal abnormal neuroplasticity in patients with psychiatric neurodevelopmental conditions. However, to the best of our knowledge, the modulatory effect of TBS on neural plasticity has never been investigated in patients with MDD. We hypothesized that patients with MDD would display decreased TBS-induced modulation of corticospinal excitability compared with healthy volunteers.

## Materials and Methods

The study was approved by a local ethics committee (CPP Sud-Est 6), ANSM 2013-A00971-44 and registered in www.Clinicaltrials.gov (NCT02438163). All patients provided written, informed consent. The trial was conducted in the Hospital Le Vinatier, University Department for treatment-resistant depression, University of Lyon, France. All patients were consecutively recruited from March 2014 to October 2017.

### Participants

Fourteen patients with unipolar MDD according to DSM 5 and 14 matched healthy controls were enrolled in the study. Three patients with MDD were not included in the final analyzed sample. One patient with MDD was excluded because of an unexpected cerebral lesion discovered during the magnetic resonance imaging (MRI); one patient withdrew her consent; one was excluded because we were not able to obtain a 1 mV baseline MEP. It should be note that one patient only took part in the cTBS session because of strong nausea on the morning of the iTBS session, and setting up another day of investigation with the patient was not possible due to the introduction of antidepressant medication. In the healthy control group, three participants were not included in the final analyzed sample: two participants withdrew their consent, and one was excluded because she presented with an antecedent of a major depressive episode. Therefore, the final analyzed sample consisted of 11 healthy participants, 10 patients with MDD who received cTBS and 11 patients with MDD who received iTBS.

Only right-handed patients (according to the Edinburgh Handedness Inventory) from both genders [eight females, three males, age range 28–61, mean = 44.6 (standard deviation = 10.8) years old] with a Montgomery–Åsberg Depression Rating Scale (MADRS) score between 20 and 35 and free from any psychotropic drugs (including antipsychotic, antidepressant, and antiepileptic drugs) were included. For patients with MDD under psychotropic drugs, the wash out period was at least of five half-life time of the concerned drugs. Exclusion criteria consisted of (i) melancholic features; (ii) presence of a neurological or psychiatric comorbidity, except for anxiety disorder; (iii) pregnancy; and (iv) contraindications for TMS.

The group of healthy controls was composed of 11 right-handed individuals [seven females, four males, age range 26–59, 42.3 (9.4) years old]. The inclusion criteria consisted of the following: (i) no current psychiatric, neurologic or infectious disease with a potential effect on the brain; and (ii) free from any psychotropic drug. The exclusion criteria consisted of (i) pregnancy; and (ii) contraindications for TMS. Further characteristics of the participants are given in Table 1.

**Table 1:** Demographic and clinical characteristics of the participants.

	Patients with MDD	Healthy controls	*p*
*n*	11	11	
Gender (female/male)	8/3	7/4	1
Age	44.6 (10.7)	42.3 (9.4)	0.59
Number of prior episodes	1.6 (1.4)	0 (0)	<0.001
MADRS	29.8 (4.7)	0 (0)	<0.001
Duration of illness (months)	19.1 (22.6)	0 (0)	<0.001
STAI trait	55.5 (10.2)	34.8 (6.6)	<0.001
STAI state before iTBS	52.4 (11.1)	27.4 (7.0)	<0.001
1 mV MEP before iTBS	59.8 (13.9)	57.5 (8.2)	0.65
AMT before iTBS	33.9 (9.1)	35.5 (7.3)	0.67
STAI state before cTBS*	54.5 (13.4)	28.5 (7.6)	<0.001
1 mV MEP before cTBS*	58.8 (12.0)	58.3 (9.5)	0.91
AMT before cTBS*	34.8 (7.6)	34.5 (6.9)	0.93

The results are given as the mean ± standard deviation. MADRS, Montgomery–Åsberg Depression Rating Scale; STAI, State-Trait Anxiety Inventory scale; AMT, activity motor threshold; 1 mV MEP, TMS intensity to obtain an MEP with a mean amplitude of 1 mV at baseline. *Only 10 patients with MDD received continuous theta-burst stimulation (cTBS).

### Transcranial Magnetic Stimulation to Assess TBS-Induced Neural Plasticity

Participants were seated in a comfortable chair with both arms supported passively. Electromyographic (EMG) recordings from the right first-dorsal interosseus muscle (FDI) were taken using Ag/AgCl surface electrodes (Disposable Surface Electrodes SEAg-C-0.7/100/22X30; Friendship Medical, Xi An, China). Raw signals were amplified and digitized using a commercially available amplifier (Keypoint Portable System). All recordings were manually analyzed offline.

TMS was applied over the left primary motor cortex (M1) using a posterior-anterior current direction through a standard figure-of-eight coil (Cool Coil Magnetic Stimulator B65, Mag2Health) connected to a MagPro-X100 stimulator. The coil was manually and tangentially placed with the handle pointing backwards at an angle of 45° to the midline. The stimulation site leading to large and stable MEPs was defined as the optimal coil position over the left M1. To ensure that the coil reliably remained over the same stimulation target throughout the entire experimental session (baseline, TBS protocol, and repeated MEP recordings), the coil was guided with an MRI-coupled neuronavigation system [SYNEIKA ONE (SYN1) version 1.5.1].

To record MEPs at baseline and repeatedly after TBS, the TMS intensity was set to evoke MEPs of approximately 1 mV (S1mV) amplitude at baseline. We measured the peak to peak amplitude of 15 MEPs at baseline and 10 MEPs (Groppa et al., 2012) at different time points: 5, 10, 20, and 30 min after the end of the TBS session.

### Theta-Burst Stimulation Procedures

**Figure 1:**
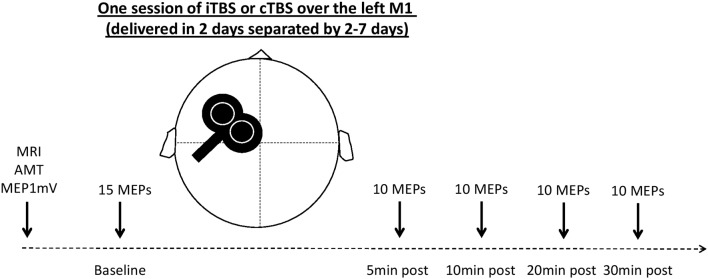
Experimental design. MRI, magnetic resonance imaging (T1-weighted image acquired to be used with the neuronavigation system); AMT, activity motor threshold; 1 mV MEP, transcranial magnetic stimulation (TMS) intensity to obtain an MEP with a mean amplitude of 1 mV; MEP, motor evoked potential; iTBS, intermittent theta-burst stimulation; cTBS, continuous theta-burst stimulation.

Participants were randomly assigned to receive two sessions of TBS delivered on two separate days. The experimental sessions were performed with a wash period between 2 and 7 days. Sessions took place at the same time of day (morning or afternoon) to prevent diurnal influences on neurophysiologic measures (Stagg and Nitsche, 2011; Kuo and Nitsche, 2012). All participants but one received one session of cTBS and one session of iTBS (Huang et al., 2005). The cTBS paradigm consisted of three pulses at 50 Hz every 200 ms for 40 s (for a total of 600 pulses). In the iTBS paradigm, participants received a 2-s train of cTBS repeated every 10 s for a total of 190 s (600 pulses). In both experiments, the intensity of stimulation was set at an intensity of 80% of the active motor threshold (AMT). AMT was assessed in the setting phase described above and was defined as the lowest intensity to obtain at least five MEPs of 200 μV over 10 stimulations in the FDI contracted at 20% of maximal strength (Huang et al., 2005). This strength was measured using a dynamometer (Hand Dynamometer Vernier HD-BTA, driven by the software Logger Pro 3); a continuous audio-visual EMG feedback was available to evaluate participants’ relaxation or their level of muscle contraction. The experimental design is illustrated in Figure 1.

### Clinical Assessments

The severity of depressive symptoms was assessed using the MADRS. State and trait anxiety levels were assessed using the State-Trait Anxiety Inventory questionnaire (STAI; Spielberger, 1989); the trait form of the questionnaire (STAI Y-B) was addressed during the inclusion visit and the state form (STAI Y-A) before each TBS session.

### Data Analysis

The sociodemographic and clinical characteristics as well as the baseline MEP measures of participants were compared between groups using independent two-tailed sample *t*-tests and Fischer’s exact tests for gender.

The relative MEP values calculated as the mean of 10 MEPs peak amplitudes post TBS/the mean of 15 MEPs peak amplitudes at baseline in each subjects were used as primary outcomes. A repeated measures ANOVA (RM-ANOVA) was undertaken with relative MEP value at the different time points as the dependent variable, group (healthy controls vs. patients with MDD) as the between-subject factor, and time as the within-subject factor. Two RM-ANOVAs were conducted to analyze the effect of iTBS on the one hand and the effect of cTBS on the other hand.

When appropriate (significant interactions in the RM-ANOVAs), *post hoc* comparisons were performed to more specifically determine the changes in MEP amplitude. TBS-induced modulation of MEP size across the five time points (baseline, 5, 10, 20 and 30 min) in both condition (iTBS and cTBS) were also investigated as the maximum peak amplitude at the individual level. Number of responders and non-responders after TBS according to Hamada et al. (2013) classification were also calculated and compared across groups using Fischer exact test. Responders and non-responders were defined according to the grand average of TBS responses below and above 1 for cTBS and iTBS, respectively (Hamada et al., 2013). SPSS 21 was used for all analyses, and the level of significance was set at *p* < 0.05.

## Results

### Sociodemographic and Clinical Characteristics

There were no significant differences in age, gender, or AMT measures between groups at baseline. State and trait anxiety were significantly higher in patients with MDD than in healthy controls (Table 1).

### TBS Induced Changes in Neural Plasticity

At baseline, there was no significant difference in the mean 1 mV MEP between the groups. Before the iTBS session, the mean amplitude of 1 mV MEP in the MDD group was 969.7 (SD 243) vs. 1055.7 (129) μV in the control group (*p* = 0.317). Before the cTBS session, the mean amplitude of 1 mV MEP in the MDD group was 1206.3 (385) vs. 975.4 (131) μV in the control group (*p* = 0.074).

**Figure 2:**
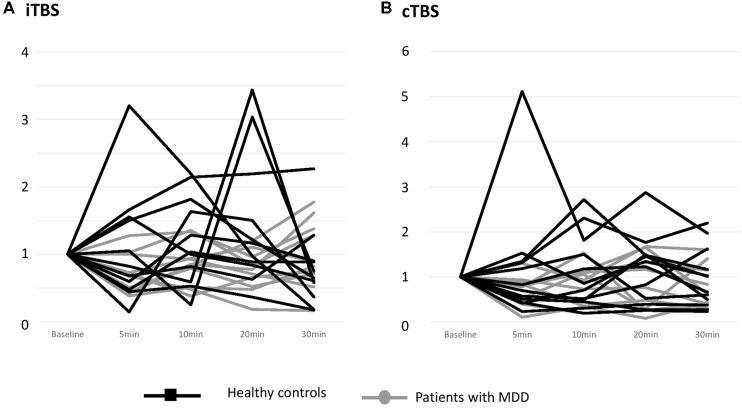
Effects of one session of TBS on MEP amplitude at an individual level. **(A)** Effects of iTBS. **(B)** Effects of cTBS. Healthy controls are outlined with dark lines; patients with major depressive disorder (MDD) are outlined with gray lines. The results are given as the mean ± SEM.

**Figure 3:**
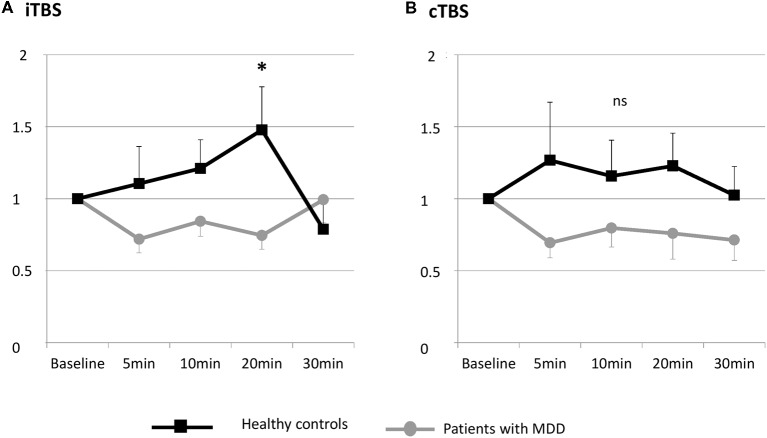
Changes in MEP amplitude after one session of TBS in patients with MDD and healthy controls at the group level. **(A)** Effects of iTBS. **(B)** Effects of cTBS. ns, not significant.

The individual data illustrating the modulation of MEP amplitudes induced by iTBS and cTBS are displayed in Figure 2. A large interindividual variability was observed in the two groups. The mean effects of iTBS and cTBS on both groups are displayed in Figure 3.

#### iTBS-Induced Changes in Neural Plasticity

The RM-ANOVA revealed a significant group × time interaction when participants were exposed to iTBS (*F*_(4,21)_ = 2.504, *p* = 0.049).

The *post hoc* comparisons revealed that after iTBS, the difference between depressed subjects and healthy controls was significant at 20 min post iTBS (*p* = 0.038). The difference was not significant at the other time points (5 min: *p* = 0.193; 10 min: *p* = 0.130; 30 min: *p* = 0.406).

Measured by the peak, the MEP size was significantly elevated by iTBS in the control group (*p* = 0.009), whereas no modulation of MEP size was induced in patients with MDD (*p* = 0.339).

**Figure 4:**
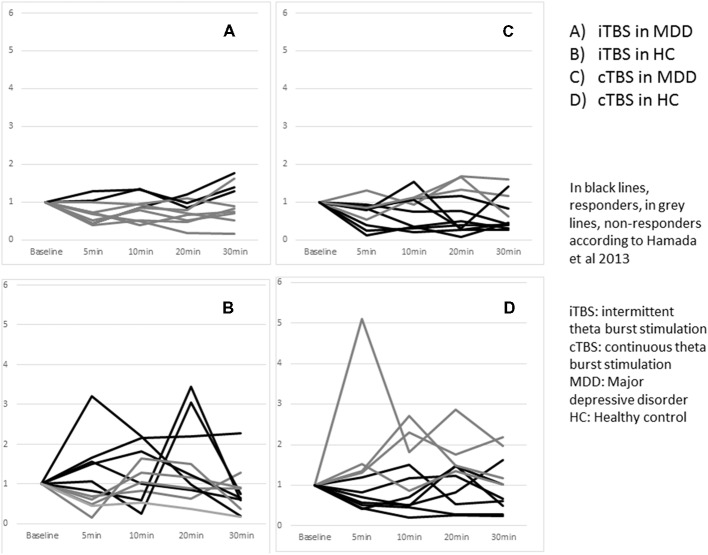
Effects of one session of TBS on MEP amplitude according to responder and non responder status across groups at an individual level.

Three patients with MDD out of the 10 were classified as responders whereas six healthy controls out of 11 were responders. The difference did not reach significance *p* = 0.39 (Figure 4).

#### cTBS-Induced Changes in Neural Plasticity

The RM-ANOVA revealed no significant group × time interaction when participants were exposed to cTBS (*F*_(4,22)_ = 0.986, *p* = 0.42).

No significant effect of cTBS on MEP measured by the peak MEP size was observed in both groups.

Eight patients with MDD out of the 11 were classified as responders whereas seven healthy controls out of 11 were responders. The difference did not reach significance *p* = 1.00 (Figure 4).

#### Safety

No serious adverse events were observed during the study. Three patients with MDD and four healthy controls reported mild headache during iTBS exposure. Two patients with MDD and one healthy control reported mild headache during cTBS exposure. This symptom disappeared after administration of a mild analgesic (paracetamol).

## Discussion

The aim of this study was to evaluate neural plasticity integrity in patients with MDD compared with that in healthy controls. Using TBS, we reported that iTBS-induced changes in neural plasticity were altered in patients with MDD. Whereas MEP size post iTBS was significantly increased in healthy controls, no effects of iTBS on MEP size were observed in patients with MDD. No effects of cTBS were observed in either group. Importantly, in addition to those effects, we observed a large interindividual variability in the effects of both iTBS and cTBS on MEP amplitude regardless of group.

The effects of rTMS protocols have been proposed to relate to activity-dependent changes in synaptic neurotransmission, reflecting neural plasticity (Ziemann et al., 2008). Among the currently available rTMS protocols, the TBS protocol has been proposed to measure neural plasticity, especially at the synaptic level (Huang et al., 2005). Depending on the stimulation parameters, TBS is assumed to induce either an inhibition of corticospinal excitability (following cTBS) or an enhancement (following iTBS). These effects outlast the stimulation period for approximately 40 min in healthy subjects (Oberman et al., 2012). The transient suppression of corticospinal excitability following cTBS and its transient enhancement following iTBS appear to be mediated by cortical processes (Di Lazzaro et al., 2011) and are assumed to reflect indexes of long-term depression (LTD) and long-term potentiation (LTP)-like mechanisms, respectively (Huang et al., 2005; Huerta and Volpe, 2009). Moreover, cTBS has been shown to involve GABAergic neurotransmission, whereas iTBS involves the glutamatergic NMDA receptor pathway (Huang et al., 2007; Stagg et al., 2009). In light of these studies, our results suggest that the LTP-like mechanisms mediated by the glutamatergic NMDA receptor pathway are impaired in patients with MDD. No significant difference was observed between patients with MDD and healthy controls regarding LTD-like mechanisms.

### Effect of iTBS on Cerebral Plasticity

The integrity of LTP-like mechanisms involving GABA and glutamatergic neurotransmission has already been investigated in patients with MDD using TMS. For instance, modulation of the duration of the interstimulus interval when applying paired-pulse TMS allows for the investigation of the inhibitory and facilitatory mechanisms mediated by GABAergic neurotransmission (short-interval intracortical inhibition–SICI, Ziemann et al., 1996) and glutamatergic neurotransmission (intracortical facilitation–ICF). Although discrepancies between studies investigating those phenomena exist, in a meta-analysis, Radhu et al. (2013) found that SICI was decreased in patients with MDD compared with that in controls. These results are in line with ours reporting alterations of neural plasticity in the motor cortex in patients with MDD. These observations are also consistent with animal studies reporting that the iTBS-induced LTP mechanisms could be modulated by the administration of GABA antagonists (Kotak et al., 2017).

#### Altered Cerebral Plasticity Following iTBS in Other Psychiatric Conditions

Our results in healthy controls are in line with the classically described effects of iTBS on MEP amplitude (Huang et al., 2005). Our results are also consistent with a previous study evaluating iTBS-induced neural plasticity in patients with psychiatric conditions. For instance, in a controlled study, Suppa et al. (2014) reported that healthy subjects displayed an increase in MEP amplitude after iTBS, whereas MEP amplitude remained unchanged in patients with Gilles de la Tourette syndrome. In the same study, the same group of authors also assessed the effect of iTBS on MEP amplitude in patients with obsessive compulsive disorder (OCD) and reported that iTBS induced an equal increase in MEP amplitude in both groups (Suppa et al., 2014). Finally, Oberman et al. (2012) reported that the iTBS-induced effects on MEP amplitude were significantly greater and longer lasting in patients with autism spectrum disorder than in healthy controls. Altogether, these results illustrate the usefulness of iTBS in revealing impaired neural plasticity in patients with psychiatric conditions, allowing us to distinguish patients with decreased iTBS-induced neural plasticity (MDD, Gilles de la Tourette syndrome), increased iTBS-induced neural plasticity (autism spectrum disorder) or similar iTBS-induced neural plasticity (OCD) compared to that in healthy controls.

### Effect of cTBS on Cerebral Plasticity

We observed that cTBS did not modulate MEP amplitude in patients with MDD. These results were in line with several studies revealing no effects of cTBS on cerebral plasticity in patients with other psychiatric conditions. For instance, no effect of cTBS on MEP amplitude was reported in patients with schizophrenia (Hasan et al., 2015), in patients with obstructive sleep apnea (Opie et al., 2013), and in patients with Gilles de la Tourette syndrome (Suppa et al., 2014). A possible explanation is that cTBS may be less efficient at inducing cerebral plasticity in patients with psychiatric disease than iTBS.

In the current study, cTBS also had no effect on MEP amplitude in healthy controls. Although these results were unexpected, they are in line with several studies showing that the effects following different TBS paradigms are subject to high interindividual variability (McAllister et al., 2009; Todd et al., 2009; Goldsworthy et al., 2012; Hamada et al., 2013). For instance, in their study investigating the effect of cTBS in patients with schizophrenia, Hasan et al. (2015) did not report any significant changes on MEP amplitude following TBS in the control group. The current results were however not in line with findings from Oberman et al. (2012) showing longer cTBS response in patients with autism spectrum disorder than in controls. The lack of a significant effect of TBS in the current study suggests that high interindividual variability can mask a significant TBS effect at the group level. However, the size of our sample did not allow us to cluster participants into TBS responders and nonresponders to explore this question.

### Strengths and Limitations

In the current study, only 10 MEPs were recorded to assess TBS-induced neuroplasticity. This could have hampered the reliability of the reported results and contribute to the observed high interindividual heterogeneity. Indeed a recent study indicated that 21 MEPs are required for reliable estimation of the MEP amplitude (Chang et al., 2016).

The lack of detailed neurocognitive assessment (allowing to detect a mild cognitive impairment which is a common finding in MDD), the lack of a preliminary evaluation of the integrity of the cortico-spinal conductivity and the lack of a more accurate T2-MRI scan instead of only the T1-weight MRI (allowing to detect brain lesions in both white and gray matter for differential diagnosis) did not allow us to exclude that such comorbidities in our sample may have influenced current results.

Further studies should investigate the close relationship between depressed mood and cognitive dysfunction since this aspect has crucial implications in determining changes of cortical excitability to TMS (Guerra et al., 2015), that can induce neuroplastic phenomena at the level of M1 (Pennisi et al., 2015) and enhance the risk of clinical deterioration in depressed subjects, in depressed subjects, especially in subjects with vascular depression (VD; Pennisi et al., 2016).

Another limit is that we only assessed the TBS-induced plasticity on the dominant M1 and not on both sides. Indeed, given that several studies found an interhemispheric difference of motor threshold it would have been interesting to evaluate cortical excitability from both hemispheres, in order to obtain bilateral data to compare before and after cTBS/iTBS.

From a more cognitive perspective, it would have been of interest to assess neural plasticity induced in the dorsolateral PFC (DLPFC), a brain region critically involved in the pathophysiology of MDD (Concerto et al., 2015). In line with this, combining EEG and TMS, Noda et al. (2018) reported an impaired neuroplasticity in the DLPFC of patients with MDD compared to healthy subjects.

Lastly, an important limitation of the current study was the relatively small sample size of included patients, which might hide significant differences between healthy subjects and patients with MDD at the group level. Nevertheless, our sample size is within the range of other TBS studies in patients with neuropsychiatric conditions (Eggers et al., 2010; Huang et al., 2011; Hasan et al., 2015). A second limitation was in the single-blind procedure of the study and the lack of a sham TBS group. Indeed, in order to not leave patients without treatment for a too long period of time, we decided to perform only two measurements separated by 2–7 days: 1 day with iTBS, 1 day with cTBS. Added a sham arm or added an arm investigating DLPFC plasticity would have increase the time where patients did not received medication.

Despite these limitations, the main strength of our study is that the included patients with MDD were drug free. Indeed, medication and especially psychopharmacological drugs are known to highly influence the cortical excitability parameters assessed by TMS (for a review see Paulus et al., 2008); therefore, this bias did not influence the current results.

## Conclusion and Perspectives

In summary, iTBS-induced cerebral plasticity was altered in patients with MDD, whereas no effect of cTBS-induced cerebral plasticity was observed. These results suggested abnormal LTP-like plasticity mediated by glutamatergic neurotransmission in patients with MDD. These abnormalities should be considered an endophenotype biological marker of MDD. However, because of the small sample of the current study, results should be taken with caution and further studies are needed to explore this topic more thoroughly. Moreover, although MDD and the so called VD share some clinical similarities, VD may rely on distinct pathophysiological mechanisms (Concerto et al., 2013) that could be highlighted by distinct neuroplasticity alterations. In the perspective of a differential diagnosis, it would be of interest to replicate our experimental protocol in the sample of VD patients. Lastly, as TBS-induced neuroplasticity results in a large interindividual variability, other TMS paradigm such as quadripulse stimulation (QPS) that have showed less inter-subject variability in healthy controls (Nakamura et al., 2016) could be useful to evaluate alteration in patients with psychiatric condition.

## Data Availability

The datasets for this manuscript are not publicly available because Data are available on reasonable request. Requests to access the datasets should be directed to philippe.vignaud@ch-le-vinatier.fr.

## Author Contributions

PV, EP and JB contributed to the conception and design of the study. PV and CD organized the database and acquired data. PV and JB performed the statistical analysis. PV wrote the first draft of the manuscript. All authors contributed to the manuscript revision, read and approved the submitted version.

## Conflict of Interest Statement

The authors declare that the research was conducted in the absence of any commercial or financial relationships that could be construed as a potential conflict of interest.
